# Withholding parenteral nutrition during the first week of critical illness increases plasma bilirubin but lowers the incidence of cholestasis and gallbladder sludge

**DOI:** 10.1186/cc12192

**Published:** 2013-03-19

**Authors:** YM Vanwijngaerden, L Langouche, M Gielen, Y Debaveye, M Casaer, C Liddle, S Coulter, R Brunner, P Wouters, A Wilmer, G Van den Berghe, D Mesotten

**Affiliations:** 1 University Hospitals KU Leuven, Belgium; 2 University of Sydney, Australia

## Introduction

Cholestatic liver dysfunction (CLD) during critical illness, defined by hyperbilirubinemia, often occurs and is associated with poor outcome. Parenteral nutrition (PN) is assumed to aggravate CLD. However, hyperbilirubinemia more frequently occurred when the start of PN was delayed until day 8 in the ICU (late PN) [1]. Late PN accelerated recovery as compared with early initiation of PN.

## Methods

This was a preplanned subanalysis of a large randomized controlled trial on early versus late initiation of PN (*n = *4,640) [1]. Plasma total bilirubin was quantified in all patients daily while in the ICU. Liver enzymes ALT, AST, GGT and ALP were quantified twice weekly in all patients while in the ICU. In a random predefined subset of patients, circulating bile salts were also quantified with MS-HPLC at baseline and on day 3, day 5 and the last day in the ICU (*n = *280). Gallbladder sludge was evaluated by ultrasound on ICU day 5 by blinded assessors (*n = *776).

## Results

From day 1 after randomization until the end of the 7-day intervention window, plasma bilirubin was higher in the late PN than in the early PN group (all *P <*0.001). In the late PN group, as soon as PN was started on day 8, plasma bilirubin also fell and the two groups became comparable. Maximum levels of GGT, ALP and ALT during the ICU stay were higher in the early PN group (all *P <*0.01). Compared with baseline, the circulating glycine and taurine conjugated primary bile salts were elevated on day 3, day 5 and last day of the ICU stay (*P <*0.01 for all). However, there was no difference between the two groups. More patients in the early PN than in the late PN group had gallbladder sludge on day 5 (45% vs. 37%; *P *= 0.04).

## Conclusion

Tolerating substantial caloric deficit by withholding PN until day 8 of critical illness increased circulating levels of bilirubin but reduced the occurrence of gallbladder sludge and lowered GGT, ALP and ALT levels. These results suggest that hyperbilirubinemia during critical illness dies not necessarily reflect cholestasis and instead may be an adaptive response. Additional analyses on a propensity score-matched patient population are ongoing.

## References

[B1] Casaer N Engl J Med 2011 365 506 516 10.1056/NEJMoa1102662 21714640

